# 

**Published:** 1869-03-15

**Authors:** 


### CONTENTS.

ORIGINAL COMMUNICATIONS:
Analysis of Hot Springs, Arkansas........ 161
Cases of Malformation of the Eye and Ear—Anophthalmos........ 163
CORRESPONDENCE........ 167
PROCEEDINGS OF SOCIETIES:
American Medical Association........ 173
Delegates to the American Medical Association........ 175
Illinois State Medical Society........ 176
Carroll County Medical Society........ 176
EDITORIAL:
A New Defence for Murder........ 178
Location for Practice........ 179
Urethral Sounds—Paper Collars—Back Volumes........ 180
LOOT:
A Practical Point in the Treatment of Throat Diseases—The Case of Dr. Earle........ 181
Prof. Halford's Treatment of Snake Bites........ 182
Strength of Carbolic Acid Solutions........ 184
Sea-Sickness........ 185
Wombless Women........ 186
India-Rubber Sponge........ 188
BOOKS RECEIVED:
Pathological Phenomena Generalized. By H. Backus, Montevallo, Ala... 189
Constitution and By-Laws of the Gynæcological Society, Boston........ 190
Treatise on Svapnia, or Purified Opium. By J. M. Bigelow, of Detroit........ 191
On the Identity of the White Corpuscles of the Blood with the Salivary Pus and Mucous Corpuscles. By J. G. Richardson, M. D........ 191
A History of the Medical Department of the University of Pennsylvania. By Joseph Carson, M. D........ 191
Pennsylvania Hospital Reports—Vol. 2........ 192
A Treatise on Diseases of the Ear, etc. By Anton Von Trolsch, M. D........ 192
A Treatise on the Diseases of Infancy and Childhood........ 192
Syphilis and Local Contagious Disorders. By J. Lewis Smith, M. D........ 192
The Part taken by Nature and Time in the Cure of Diseases, etc........ 192
ERRATA........ 192

